# Supplementary figures and images for: Genome-Wide Identification and Characterization of the Oat (Avena sativa L.) WRKY Transcription Factor Family
Source: Genes (Basel). 2022 Oct 21;13(10):1918. doi: 10.3390/genes13101918 (PMC9601435; doi:10.3390/genes13101918)

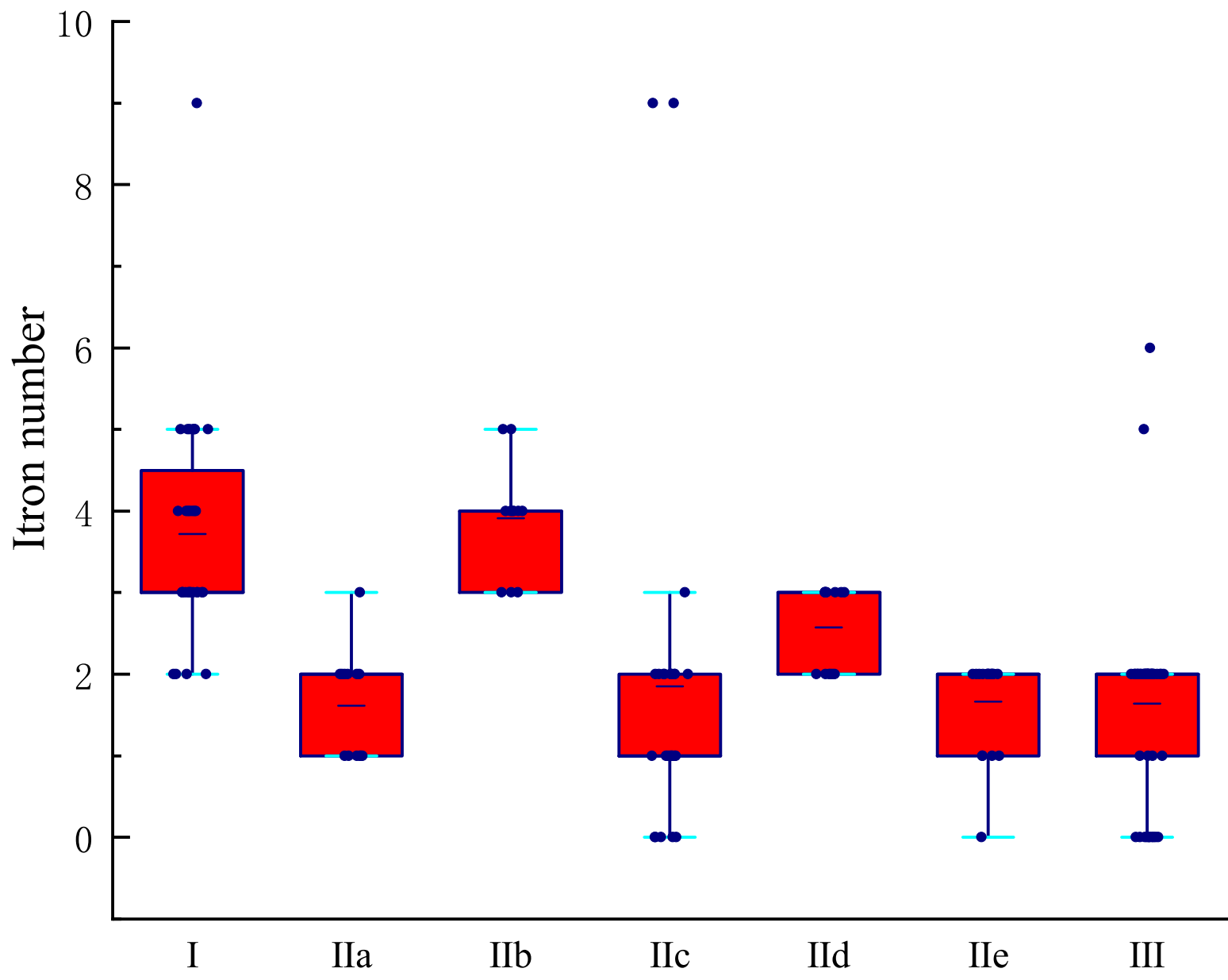

Supplement: Supplementary file 1 [file genes-13-01918-s001.zip › Figure S1.pdf]

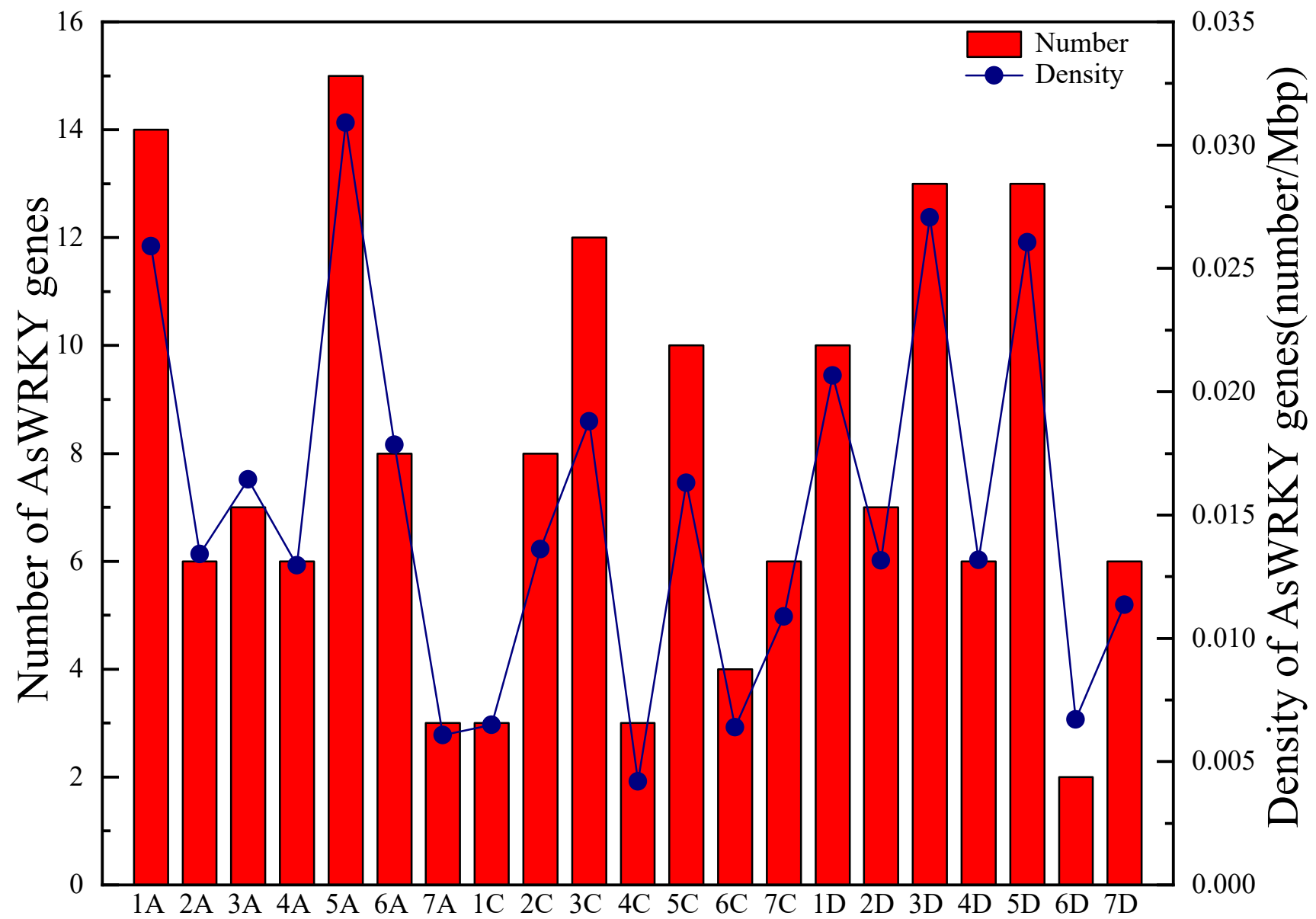

Supplement: Supplementary file 1 [file genes-13-01918-s001.zip › Figure S3.pdf]

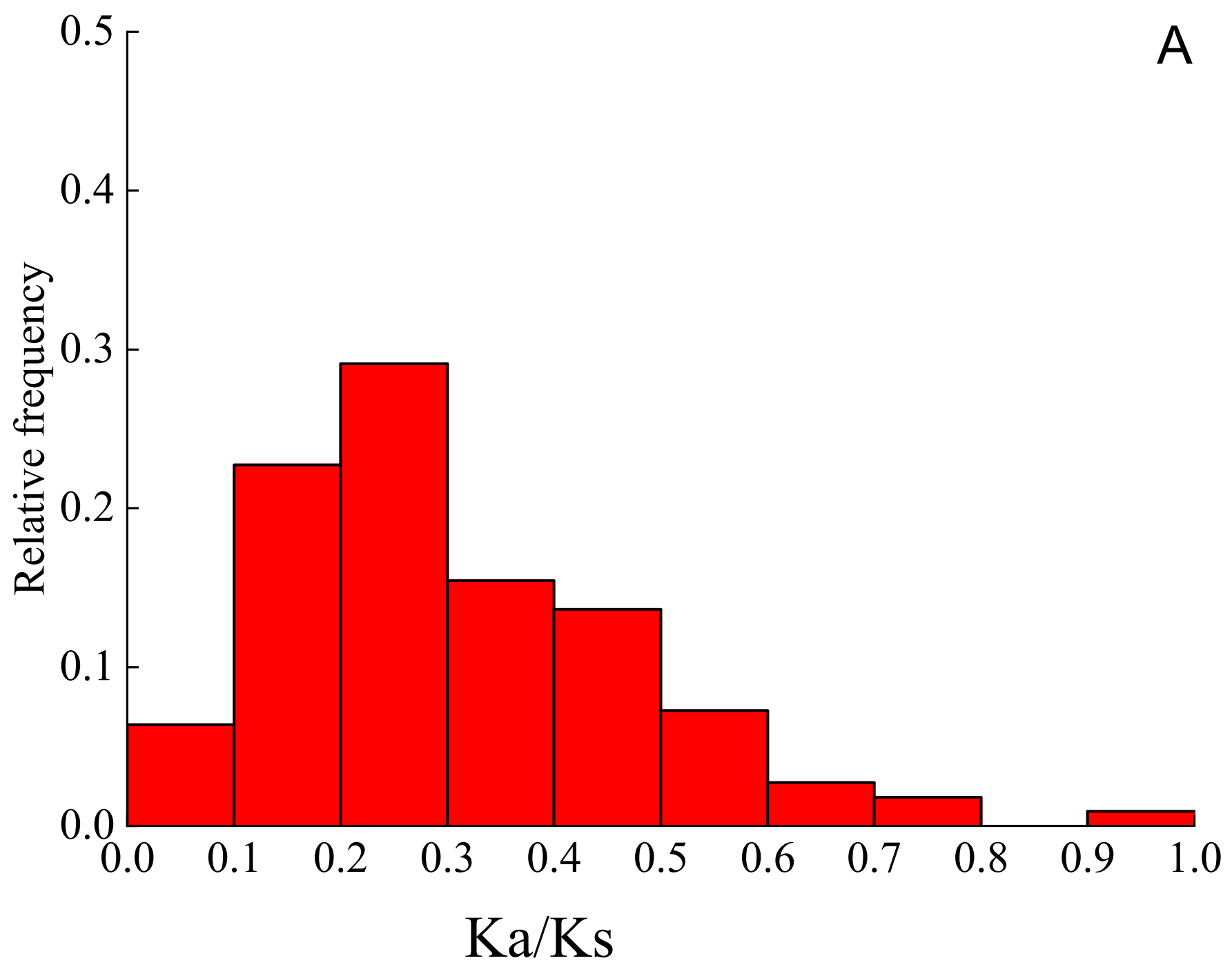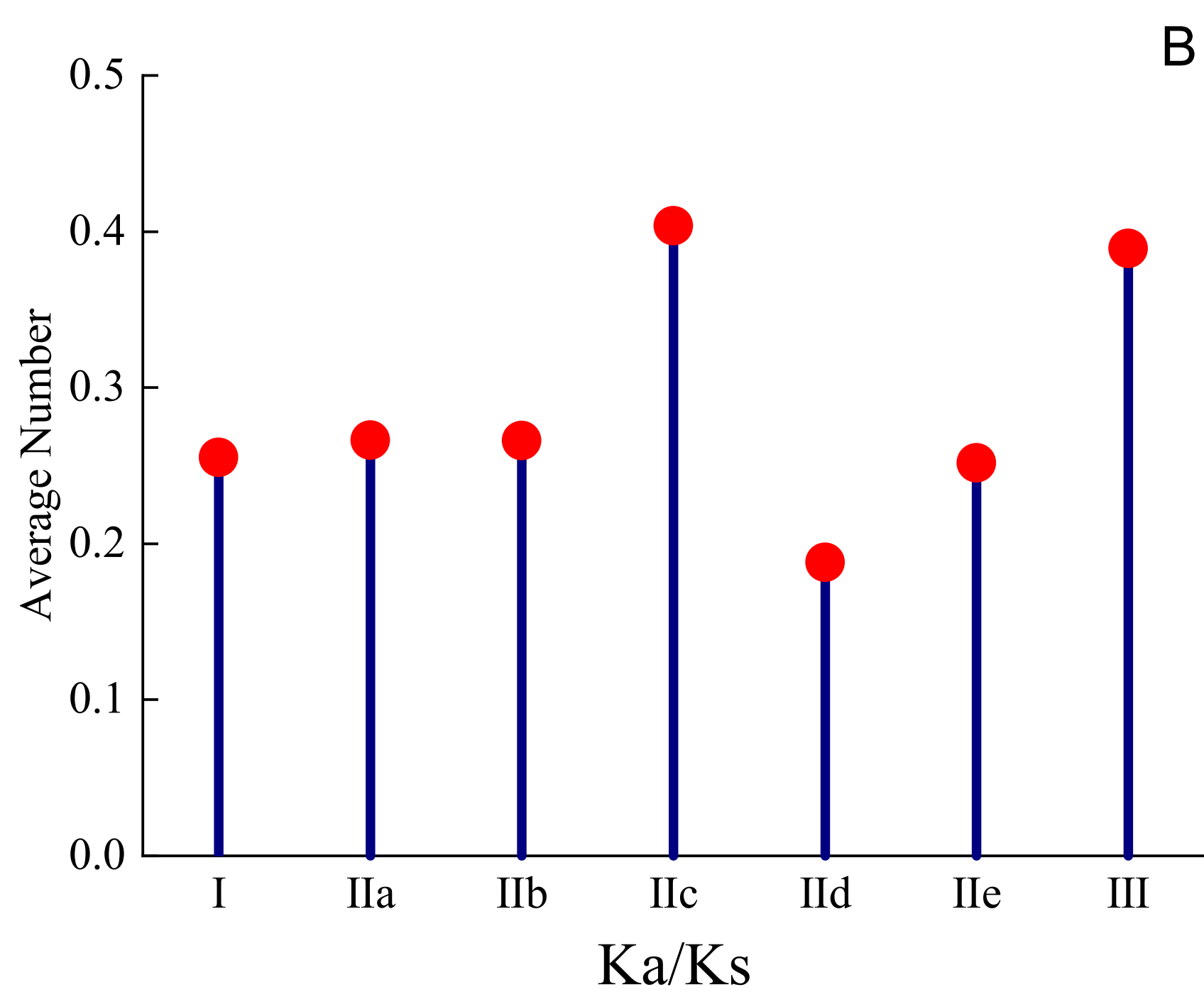

Supplement: Supplementary file 1 [file genes-13-01918-s001.zip › Figure S4.pdf]

GO Term

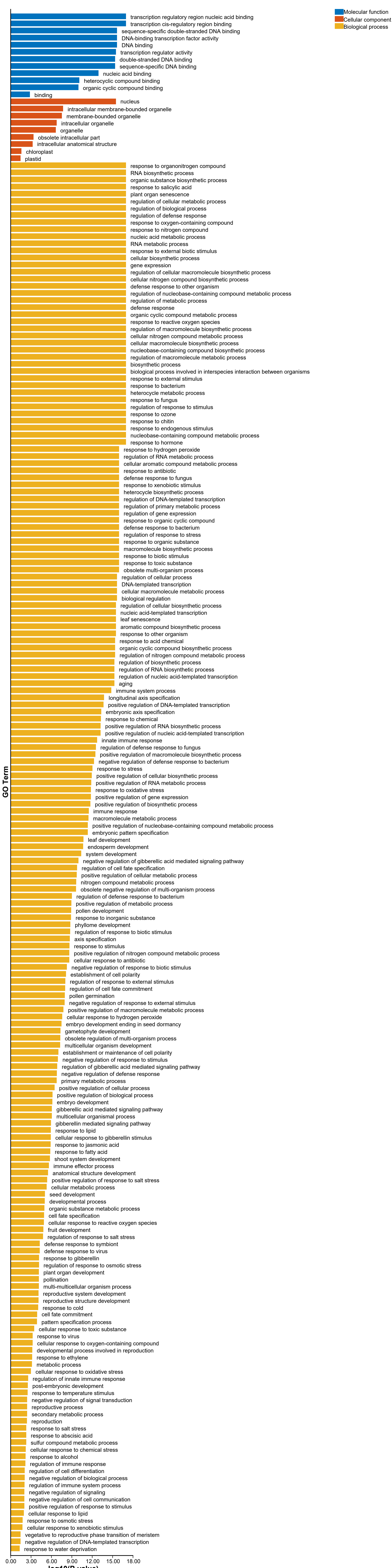

Supplement: Supplementary file 1 [file genes-13-01918-s001.zip › Figure S5.pdf]

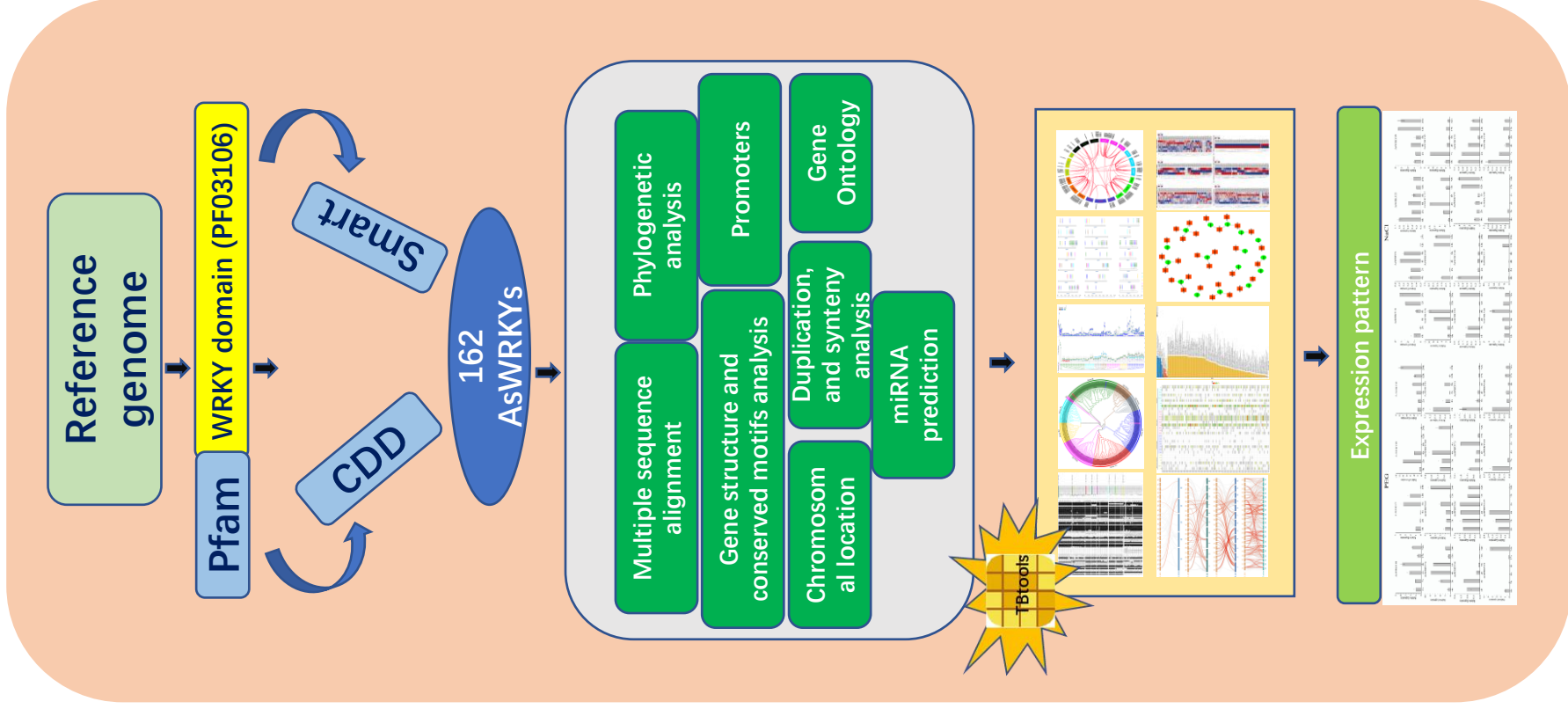

Supplement: Supplementary file 1 [file genes-13-01918-s001.zip › Figure S6.pdf]
