# Supplementary material for: Genome-Wide Identification and Characterization of the Oat (Avena sativa L.) WRKY Transcription Factor Family
Source: Genes (Basel). 2022 Oct 21;13(10):1918. doi: 10.3390/genes13101918 (PMC9601435; doi:10.3390/genes13101918)
Supplement: Supplementary file 1 [file Figure S2.pdf › Figure S2.pdf]

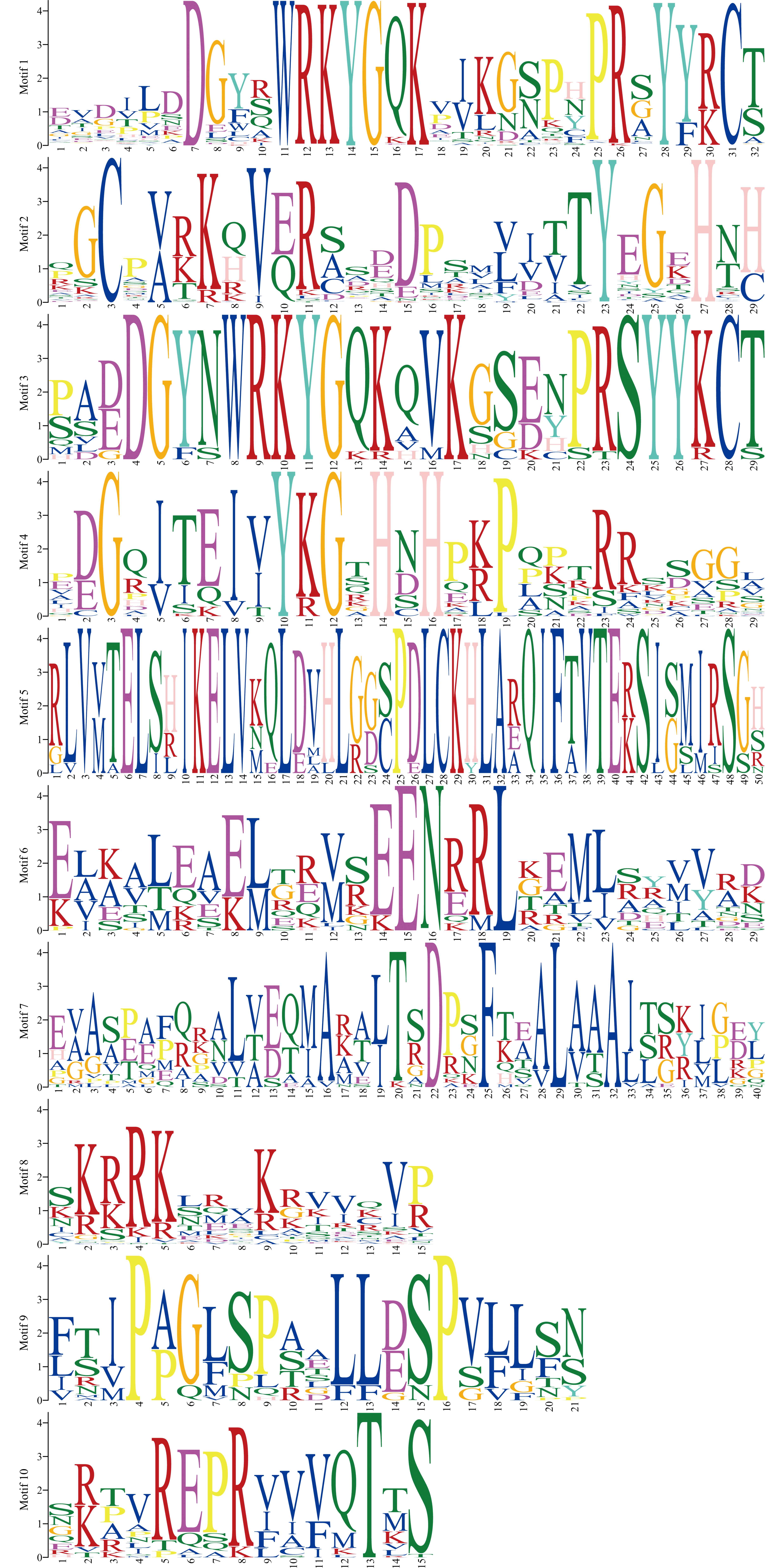
Motif 1
Motif 2
Motif 3
Motif 4
Motif 5
Motif 6
Motif 7
Motif 8
Motif 9
Motif 10
